# Impact of Medication Adherence on Bone Mineral Density and Fracture Risk in Patients With Osteoporosis: A Systematic Review

**DOI:** 10.7759/cureus.42115

**Published:** 2023-07-19

**Authors:** Manea M Alahmari, Ali I AlHilali, Taef A Thabet, Mushabab A Alshahrani, Wejdan A Mobasher, Dalia A Al Mubarak, Abdullah M Alshamrani, Raghad S Gohman, Seham A Alqarni, Malak M Alqahtani

**Affiliations:** 1 Department of Endocrinology and Diabetes, Muhayel General Hospital, Muhayel, SAU; 2 Department of Endocrinology and Diabetes, Aseer Central Hospital, Abha, SAU; 3 Department of Internal Medicine, Aseer Central Hospital, Abha, SAU; 4 Department of Internal Medicine, Prince Faisal bin Khalid Cardiac Center, Abha, SAU; 5 Department of Endocrinology and Diabetes, Armed Forces Hospital Southern Region, Khamis Mushait, SAU

**Keywords:** medication, risk, fracture, osteoporosis, adherence

## Abstract

Osteoporosis is a chronic, prevalent disease marked by decreased bone mass and changes in bone anatomy associated with significant morbidity. The management of osteoporosis necessitates long-term therapy for which patient adherence is of vital importance. In the present review, we aim to collect all potential evidence from relevant studies that reported the impact of medication adherence on bone mineral density and fracture risk in patients with osteoporosis. We have conducted both electronic and manual search strategies within the potential databases and included articles and reviews to find relevant studies. We have assessed the effects of osteoporotic medication adherence on fracture rates and bone mineral density. The study participants were divided into two groups, adherent and non-adherent. Studies from the year 2010-2023 were included. Final inclusion consisted of 14 studies that showed variation in adherence rates with only three studies reporting optimal adherence followed by two studies with nearly half adherent population while the rest of the studies reported low medication adherence. The highest adherence rate reported was 82% while the lowest was 8%. Among the included studies the fracture rates varied significantly. Decreased rates of fracture were observed in the adherent population however two of the included studies were contrary to these findings. Additionally, only three studies discussed the effect of adherence on bone mineral density. Lack of medication adherence is linked to an increased risk of fracture, and low bone mineral density, further associated with more severe complications as per the evidence from the literature. However, variation in the fracture rates as observed in our findings advocates the need for further research for the generalizability of results.

## Introduction and background

Osteoporosis is a chronic skeletal disease characterized by low bone mass and degradation of bone tissue microstructure, leading to enhanced bone fragility and a higher susceptibility to fractures. The frequency of osteoporosis-related fractures increases in women after the age of 55 and in men after 65 [1]. Osteoporosis affects 200 million people globally, with osteoporotic fractures accounting for nearly 8.9 million fractures annually. With the global population aging, the prevalence of osteoporosis is expected to rise dramatically in the coming years [2]. The Eastern Mediterranean region reports that almost 24.4% of its population is affected by osteoporosis. A particularly high prevalence of 32.7% has been observed in Saudi Arabia, whereas in Kuwait, the prevalence stands at 15.1% [3].

Osteoporosis can result in heightened hospitalization rates due to fractures and other related complications. It's noteworthy that only one-third of fracture patients manage to regain their previous level of functionality [4,5]. Osteoporotic fractures predominantly affect the hip, vertebrae, and distal forearm, leading to high morbidity and mortality rates, as well as a diminished quality of life. This is largely due to the increased incidence of co-existing conditions these patients experience alongside fractures. Hence, the primary aim of osteoporosis treatment is to minimize fracture risk [2]. Nonadherence to pharmacological treatment presents the most common challenge in managing osteoporosis. Like many chronic diseases, poor treatment adherence for osteoporosis imposes a significant burden on both patients and healthcare systems [6]. Nonadherent individuals face severe consequences such as reduced bone mineral density response, impaired bone marker suppression, and an increased risk of fractures. Although the shift from daily to weekly oral bisphosphonate doses has improved compliance, adherence to weekly bisphosphonate treatment remains subpar. Factors such as patients' health attitudes, inadequate patient education, and aging serve as barriers to adherence [7].

The degree to which a patient follows the recommended dose, interval, and timing of a dosing regimen is referred to as compliance while the act of continuing treatment for the allotted time frame is termed as medication persistence. Adherence is sometimes used interchangeably with compliance or as a more general term to refer to both compliance and persistence [8]. The clinical effectiveness of osteoporotic treatment is significantly associated with the medication adherence of patients. Within a year of starting an osteoporosis treatment, people who are adherent versus non-adherent can show different changes in bone mineral density additionally, greater compliance shows increasing benefits including a reduction in fracture risk [8]. Up to a 60% lower risk of hip fracture was linked to continued bisphosphonate medication. Although there is a distinct relationship between the level of adherence and the reduction of fracture risk, there are also a lot of variances among age groups and fracture types. The risk of all clinical fractures is observed to be reduced by 20-45% by high medication adherence. The beneficial effects of medication compliance can be observed with as little as 50% adherence and are even greater with at least 75-80% adherence [8]. In the present review, we aim to collect all potential evidence from relevant studies that reported the impact of medication adherence on bone mineral density and fracture risk in patients with osteoporosis.

## Review

Methods

Definition of Outcomes and Inclusion Criteria

Our objective was to examine how adhering to medication impacts bone mineral density (BMD) and fracture risk among patients with osteoporosis. The medication possession ratio, which is a measure of medication adherence, is used to compare fracture rates and BMD among adherent and non-adherent populations. We restricted our analysis to original research studies that involved adult patients with osteoporosis who were receiving pharmacological therapy. Studies lacking a non-adherence comparison group or follow-up data on medication adherence were excluded, along with case reports and case studies with small sample sizes or insufficient descriptive statistics. We also excluded non-human or laboratory studies, incomplete studies, non-original investigations, abstract-only articles, theses, protocols, and articles not published in English.

Search Strategy

After obtaining our desired outcomes, we performed a brief manual screening of potentially included studies to identify relevant keywords for the most appropriate search term. Our search terms included (osteoporosis OR bone loss OR bone density OR bone health) AND (medication adherence OR treatment compliance OR patient compliance OR drug therapy) AND (fractures, bone OR fracture risk OR patient outcomes). Database for the search included PubMed, Google Scholar, and Science Direct. Our search was restricted to the title and abstract of the search results to ensure we captured all relevant studies. All results were then saved to an Endnote library, where we identified and removed duplicates across the different databases. Additionally, we manually searched the reference lists of the included studies and relevant reviews, as well as similar article sections in PubMed, to identify any missed studies by our electronic search strategy. We followed the Preferred Reporting Items for Systematic Reviews and Meta-Analyses (PRISMA) guidelines throughout all stages of this systematic review.

Screening and Extraction

To ensure the accuracy and quality of our review process, we implemented a double screening strategy, which involved screening both titles/abstracts and full texts. Two reviewers conducted the screening process in a blinded manner, and a senior member oversaw the entire process and facilitated discussions among the reviewers in case of discrepancies. We constructed an extraction sheet that was organized in a manner relevant to our research objectives, which included baseline characteristics, publication details, abstracts, decisions to include or exclude articles, and the reasons for exclusion. We also identified whether each study was a clinical trial or not. We made sure to include all relevant articles that met our criteria.

Quality Assessment

We extracted information from the included studies regarding the potential risk of bias in these studies. To assess the quality of observational studies, we used the modified Newcastle-Ottawa scale (NOS), which comprises four domains: the quality of methods, compatibility, assessment, and reporting of outcomes/exposure. Studies were graded on a scale of 0 to 10 based on the degree of bias and marked as excellent, good, satisfactory, or not satisfactory. For the two randomized control trials included in our review, we used the Cochrane collaboration risk of bias tool to assess quality. This tool assesses the risk of bias in five domains, including randomization, missing outcome data, deviation from intended intervention, measurement of outcome, and selection of reported result. It is graded as low, moderate, or high risk of bias.

Results

Search Results

**Figure 1:**
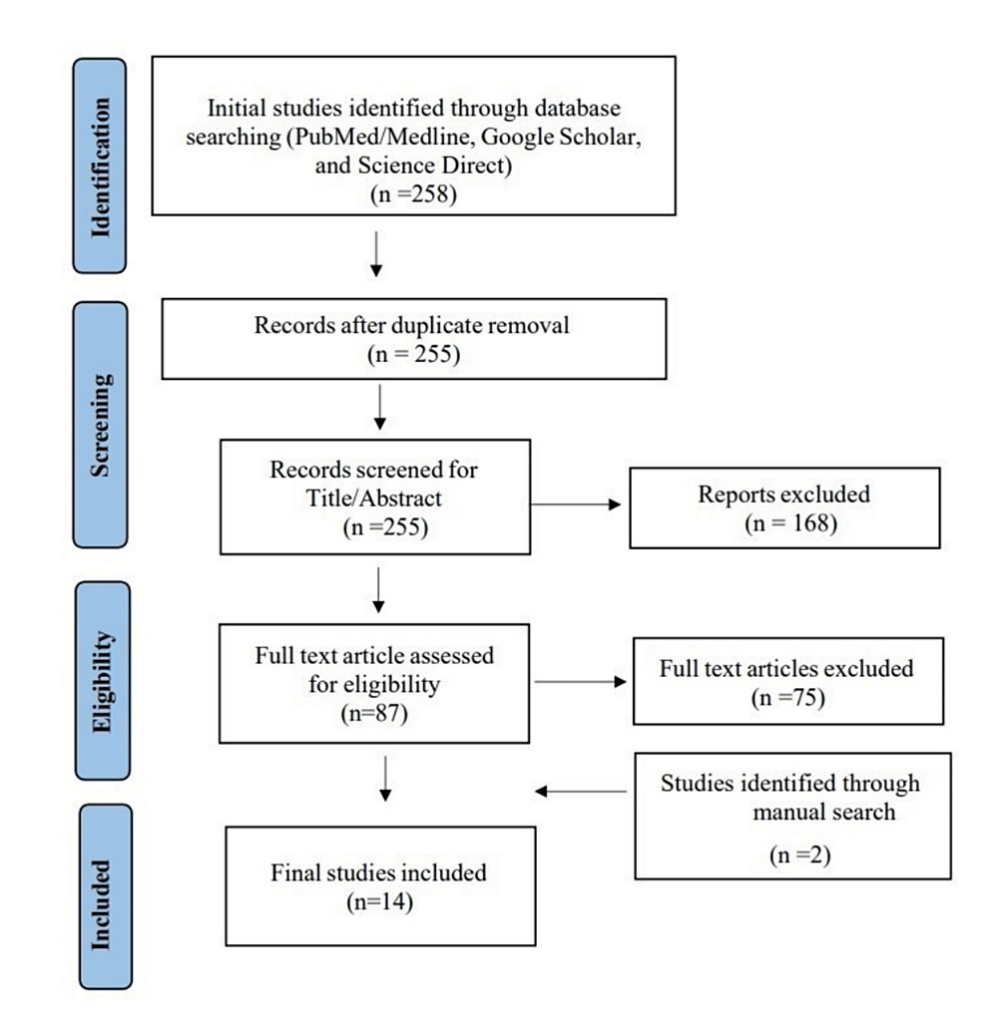
PRISMA flow chart PRISMA: Preferred Reporting Items for Systematic Reviews and Meta-Analyses

We conducted the search strategies as described above and identified a total of 258 citations, which were then reduced to 255 after removing duplicates. After screening titles and abstracts, only 87 citations were considered eligible for the next steps. Full-text screening narrowed down the number to 14 articles that matched our inclusion and exclusion criteria. Figure 1 shows the detailed search strategy and screening process.

Results of Quality Assessment

The quality assessment of the included studies revealed that the majority of studies had good quality with a low risk of bias, while only three studies had satisfactory results. None of the studies were found to have excellent or unsatisfactory results. The Cochrane collaboration risk of bias tool also showed an overall low risk of bias for the two included trials. Detailed results of the quality assessment according to the adjusted NOS and Cochrane collaboration risk of bias tool are illustrated in (Table 1 and Table 2).

**Table 1:** Summary of the results of bias assessment of the included studies using the modified Newcastle-Ottawa scale (NOS) for cross-sectional and case-control studies.

Author		Selection			Comparability	Outcome/Exposure		Total score	Quality
	Cross-sectional study								
	Representativeness of the sample	Sample size	Non-respondents	Ascertainment of the exposure	The subjects in different outcome groups are comparable	Assessment of outcome	Statistical analysis		
Cho H et al.	1	1	1	1	2	1	1	8	Good
Curtis et al.	1	1	1	1	1	1	1	7	Good
Wade, sally et al.	1	1	1	1	2	1	1	8	Good
Lakatos et al.	1	1	1	1	1	1	1	7	Good
Keshishian et al.	1	1	1	1	2	1	1	8	Good
Soong et al.	1	1	1	1	2	1	1	8	Good
Lin et al.	1	1	1	1	2	1	1	8	Good
Modi et al.	1	1	1	1	2	1	1	8	Good
Hadji et al.	1	1	1	1		1	1	6	Satisfactory
Weycker	1	1	1	1	2	1	1	8	Good
Eastell et al.	1	1	1	1		1	1	6	Satisfactory
	Case-control study								
	Definition of cases	Representativeness of cases	Selection of controls	Representativeness of controls	Comparability of cases and controls on the basis of design or analysis	Ascertainment of the exposure	The same method of ascertainment of cases and controls	Nonresponse rate	
Kim. Seihee et al.	1	1	1	1	1	1	1		Good

**Table 2:** Quality assessment of RCT using the Cochrane collaboration risk of bias tool RCT: randomized controlled trial

	D1	D2	D3	D4	D5	Overall
Curtis JR et al.	Low	Low	Low	Low	Low	Low risk of bias
Black et al.	Low	Low	Low	Low	Low	Low risk of bias
	D1	Bias arising from the randomization process				
	D2	Bias due to deviations from intended interventions				
	D3	Bias due to missing outcome data				
	D4	Bias in the measurement of outcome				
	D5	Bias in the selection of the reported result				

Characteristics of the included studies

We included 14 studies that recruited 1738483 patients and were published between 2010 and 2023. Among the total included patients 52586 were males and 1678099 were females. The majority of the studies were observational studies, two studies were randomized control trials, and two were case-control studies. Regarding the geographical distribution of the included studies almost half of the studies were from the United States followed by two studies from Korea, two from China, and each study from Hungary, Germany, and the United Kingdom. The study participants were divided into two groups adherent and non-adherent to their prescribed osteoporotic medication. Curtis et al. reported the highest adherence rate of 82% among our included studies [9] followed by Eastell et al. who described a 79% compliance rate [10] and Hadji et al. reported 66.3% population as adherent [11].

Keshishian et al. revealed around half of the study population was highly adherent (50.9%) and 16.6% were moderately adherent [12] while Modi et al. reported a 40.5% adherence rate [13] and Wade et al. stated 39.2% adherence rate [14]. Three studies reported adherence rates in terms of medication possession ratio, in a study by Lin et al. only 3412 participants had a medication possession ratio greater than 80 [15] while in Soong et al. study only 27.5% had a ratio greater than 80 [16] and Weycker et al. reported mean medication possession ratio of 0.57 [17]. One study reported an adherence rate of 22.2% [18] while a study by Black et al. reported a quite low adherence rate of 8.3% [19]. In a study by Kim et al. 50.01% of participants were compliant [20] while in P. Lakatos et al. study 33.58% were compliant [21]. All the baseline characteristics of these studies are shown in Table 3. Almost all studies had larger sample sizes except two studies which had smaller study samples comparatively which may be due to the specific design and objective.

**Table 3:** Baseline characteristics of the included studies *MPR: medication possession ratio

Author	Country	Study type	Year of publication	Study period	Sample size adherent/Non-adherent	Age (Mean or %)	Gender
Curtis JR et al.	United States	Randomized control trial	2010	4 Years	3,169 Adherent 82% Non-adherent: 17%	--	Female: 100%
Lin et al.	China	Observational	2011	3 years	8936 MPR <80 5524 MPR> 80 3412	74.0 ± 8.9	Male: 19.32% Female: 80.68%
Eastell et al.	United Kingdom	Observational	2011	1 year	1317 Compliance 79% Non-compliant 21%	71 years	Female: 100%
Hadji et al.	Germany	Database analysis	2012	3 years	4147 Adherent 66.3% Non-adherent: 33.7%	≥60 years (89.3%) < 60 years (10.7%)	Female: 100%
Curtis JR et al.	United States	Observational	2012	3 years	7,105	--	Male: 15.55% Female: 84.45%
Weycker et al.	United States	Retrospective cohort	2012	5 years	644 Mean MPR 0.57	66 years	Female: 100%
Wade, Sally W et al.	United States	Observational	2012	6 years	33,558 Adherent 39.2% Non-adherent:60.8%	59.5 years	Male: 6.34% female: 93.66%
Black et al.	United States	Randomized control trial	2013	24 months	781 Adherent 8.3% poorly-adherent: 4.9%	63.8 years	Female: 100%
Soong et al.	China	Retrospective analysis	2013	1 year	32,604 MPR< 80 72.5% MPR>80 27.5%	72.44±9.51 years	Female: 85.36% Male: 14.64%
Lakatos et al.	Hungary	Observational	2014	6 years	Compliant 33.58% Non-compliant 66.42%	70 years	Female: 100%
Modi et al.	United States	Retrospective analysis	2015	7 years	57913 Adherent 40.5% Non-adherent: 59.5%	64 years	Female: 100%
Keshishian A et al.	United States	Observational	2017	11 months	103852 Highly adherent 50.9% Moderate adherence 16.6% Low adherence 32.5%	≥ 65 years 8.0%	Female: 100%
Cho H et al.	Korea	Case-control	2018	1 year	4,38845 Adherent 22.2% Non-adherent 77.8%	66.9±8.4 years	Male: 9.05% Female: 95.95%
Kim Seihee et al.	Korea	Matched case-control	2022	1 year	45612 Adherent 50.01% Non-adherent 49.99%	--	Male 6.85% Females 76.05%

BMD measurements were performed using different methods as described in the respective studies. Eastell et al. utilized dual-energy X-ray absorptiometry (DXA) to measure lumbar spine and hip BMD values, obtained from Lunar, Norland, or Hologic densitometers. T-scores at the hip were calculated based on reference data from the Third National Health and Nutrition Examination Survey [10]. Weycker et al. extracted information from BMD scans, including BMD values and estimated T-scores, individuals had a pre-treatment T-score below -1.0 at the left total hip, indicating low bone density [17]. In Curtis et al.'s study, BMD was measured at the hip and posterior-anterior spine on all participants using Hologic QDR-2000 densitometers (Hologic, Inc., Waltham, MA). The FITI and FIT-II cohorts included women with low bone mass (defined as a T-score of less than -1.6 at the femoral neck) with and without existing vertebral fractures at baseline, respectively [9]. These methodological variations highlight the need to consider these differences when interpreting and comparing the findings of the included studies in the systematic review.

Study outcome measures

Fracture rates and BMD changes were observed and compared among adherent and non-adherent groups across the included studies. Black et al. reported a 0.90% fracture rate in the adherent group and 10.05% in the non-adherent group at a follow-up time of 24 months including 12 months follow-up without treatment [19]. In a study by Lakatos et al., the total fractures reported in the non-adherent group were 11.33% while in the adherent group, they were lower comparatively almost 9.81% [21]. Similarly, Cho et al. reported a slightly lower fracture rate in the adherent group 6.95% than in the non-adherent population (7.63%) [18] followed by findings of Soong et al. who reported a 27.5% fracture rate in adherent participants and 72.5% in non-adherent [16]. Modi et al. revealed vertebral, hip, and other site fracture rates of 0.5%, 0.2%, and 1.4% among the adherent population respectively while for the non-adherent population, the rates were 0.8%, 0.4%, and 1.7% respectively [13], a study by Lin et al. showed 24.2% hip fractures and 75.8% vertebral fractures in adherent group and 27.3% hip fractures and 72.7% vertebral fractures in the non-adherent group [15]. On the contrary, Hadji et al. reported 88.1% and 85.0% fracture-free, respectively; p=0.0147 [11]. Additionally, among the adherent population with a medication possession ratio >80, the fracture rates reported by Curtis et al. for hip, vertebral, and wrist fractures were 1.83%, 3.09%, and 6% respectively while for non-adherent participants with a medication possession ratio <80 the fracture rates for these sites were 0.57%,0.66%, and 0.66% respectively [9]. Four authors divided the adherent study population into three categories: high, moderate, and low adherence. Keshishian et al. revealed 4.8% of all fractures in the high adherent population and 7.6% of all fractures in low adherent participants [12] while compared with women with lower placebo compliance, bone loss at the total hip was lower in compliant placebo-treated women (-0.43%/year versus -0.58%/year) [22]. Wade et al. reported 31.3% of vertebral fractures in the high adherent group and 53.1% of vertebral fractures in the low adherent group [14]. Kim et al. revealed 36.29% fractures among high adherent participants with a medication possession ratio greater than 90 and 37.11% fractures in the low adherent study population [20]. Regarding the effect of medication adherence on BMD only three studies discussed it, with Curtis et al. reporting 0.58±0.07 BMD of femoral neck in non-adherent population and 0.58±0.06 in adherent population [9] while Weycker et al. reported mean unadjusted change in BMD was -0.8%, 0.7%, 2.1%, 2.1%, and 2.9% at equal intervals of medication possession ratio [17] and Eastell et al. stated that increased median BMD changes were observed among adherent participants [10]. These results are elaborately explained in (Tables 3a-3b). These results are elaborately explained in Tables 4A-4B and Table 5.

**Table 4:** 4A: Summary of the outcomes of the included studies in this review (non-adherent/MPR <80 vs. adherent/MPR>80). 4B: Summary of the outcomes of the included studies in this review (high adherence (MPR ≥ 80%) vs. moderate adherence (50%≤ MPR < 80 vs.80%) vs. low adherence (MPR < 50%)) MPR: medication possession ratio

Author	fracture rate		Follow up
	Non-adherent/MPR <80	Adherent/MPR>80	
Curtis JR et al.	Hip: 18 (0.57%)	Hip: 58 (1.83%)	12.8 months
	Vertebral: 21 (0.66%)	Vertebral: 98 (3.09%)	
	Wrist: 24 (0.66%)	Wrist: 190 (6%)	
Black et al.	Before treatment: vertebral fractures: 66 (19.5%)	Before treatment: vertebral fractures: 73 (16.5%)	24 months
	Vertebral fracture: 34 (10.05%) (24 months, and a follow-up of 12 months with no treatment)	Vertebral fracture: 6 (0.90%) (24 months, and a follow-up of 12 months with no treatment)	
Lakatos et al.	Total fractures: 1,936	Total fractures: 847	Not reported
	Hip: 380	Hip: 115	
	Spine: 201	Spine: 90	
	Wrist: 887	Wrist: 430	
	Other: 690	Other: 296	
Cho H et al.	Total: 26,057 (7.63%)	6,771 (6.95%)	Not reported
	Hip: 2,179 (8.4%)	Hip: 609 (9%)	
	Vertebral: 17,744 (68.1%)	Vertebral: 4,896 (72.3%)	
	Wrist: 4,558 (17.5%)	Wrist: 950 (14%)	
	Humerus: 779 (3%)	Humerus: 185 (2.7%)	
	Multi fractures: 797 (3.1%)	Multi fractures: 131 (1.9%)	
Soong et al.	Re fracture: 871 (72.5%)	Re fracture: 330 (27.5%)	3 years
Lin et al.	Hip: 1,508 (27.3%)	Hip: 826 (24.2%)	4 years
	Vertebral: 4,016 (72.7%)	Vertebral: 2,586 (75.8%)	
Modi et al.	Vertebral: 279 (0.8%)	Vertebral: 115 (0.5%)	2 years
	Hip: 145 (0.4%)	Hip: 57 (0.2%)	
	Others: 590 (1.7%)	Others: 320 (1.4%)	
Hadji et al.	Total fracture: 15%	Total fracture: 11.9%	2 years

Author	fracture rate			Follow up
	High Adherence (MPR ≥ 80%)	Moderate Adherence (50%≤ MPR < 80%)	Low Adherence (MPR < 50%)	
Keshishian A et al.	All fractures: 677 (4.8%)	All fractures: 402 (8.7%)	All fractures: 686 (7.6%)	1 year
	Hip/pelvis/femur fractures: 311 (45.9%)	Hip/pelvis/femur fractures: 214 (53.2%)	Hip/pelvis/femur fractures: 384 (56%)	
	Clinical vertebral fractures: 195 (28.8%)	Clinical vertebral fractures: 98 (24.4%)	Clinical vertebral fractures: 162 (23.6%)	
	Other non-vertebral fractures: 174 (25.7%)	Other non-vertebral fractures: 93 (23.1%)	Other non-vertebral fractures: 143 (20.9%)	
Curtis JR et al.	Major osteoporotic fracture: 227	Major osteoporotic fracture: 107	Major osteoporotic fracture: 268	Not reported
	Hip fracture: 93	Hip fracture: 50	Hip fracture: 95	
Wade, Sally W et al.	Vertebral fracture: 50 (31.3%)	Vertebral fracture: 25 (15.6%)	Vertebral fracture: 85 (53.1%)	>12 months
	Hip fracture: 28 (31.5%)	Hip fracture: 9 (10.1%)	Hip fracture: 52 (58.4%)	
	Others: 248 (36.2%)	Hip fracture: 104 (15.2%)	Hip fracture: 334 (48.7%)	
Kim Seihee et al.	MPR ≥ 90	MPR ≥ 70	MPR ≥ 50 [22]	Not reported
	Vertebral fracture: 12364 (36.29%)	Vertebral fracture: 8423 (36.94%)	Vertebral fracture: 5670 (37.11%)	

**Table 5:** Summary of the Bone Mineral Density outcome of the included studies in this review MPR: medication possession ratio, BMD: bone mineral density

Author	Bone Mineral Density (BMD)		Follow up
	Non-adherent/MPR <80	Adherent/MPR>80	
Curtis JR et al. [23]	Femoral neck: 0.58 ± 0.07	Femoral neck: 0.58 ± 0.06	Not reported
	Posteroanterior spine: 0.82 ± 0.13	Posteroanterior spine: 0.83 ± 0.14	
Weycker et al. [20]	Within the five equi-intervals of MPR, mean unadjusted BMD change [95% CI]: -0.8% [-1.6, 0.1], 0.7% [-0.3, 1.7], 2.1% [1.1, 3.0], 2.1% [1.4, 2.9], and 2.9% [2.3, 3.5]. In adjusted analyses, BMD% change was higher (1.4-3.4; p<0.05 for all) in the highest four MPR intervals respectively vs. lowest MPR interval		27.1 months (mean)
Eastell et al. [21]	BMD response was associated with adherence for both spine and hip BMD p < .0001 respectively greater median bmd changes observed with higher medication adherence		1 year

Discussion

To achieve the best therapeutic results with osteoporosis medication, maintaining adherence is essential. However, prior research on anti-resorptives, primarily bisphosphonates, revealed that at one year, more than half of all patients did not adhere to or continue taking their drug regimens [23]. Treatment for osteoporosis has generally been proven to enhance patients' health status by lowering their risk of both vertebral and non-vertebral fractures. Despite this, only a small percentage of people with osteoporosis are now receiving treatment to prevent fractures. In addition to the lack of treatment, poor adherence to osteoporotic treatment in clinical practice has also been described. Less than half of osteoporotic patients adhered to the prescribed treatment and a significant fraction of them stopped within 30 days, according to findings of a number of studies. The long-term nature of osteoporosis therapy and the patient's delayed recognition of its benefits could both contribute to poor adherence. However, non-adherent osteoporotic patients have a higher risk of subsequent fracture, which is quite interesting given that those who have previously suffered a fragility fracture have a higher risk of subsequent fracture development than those who have not [24].

Medication adherence for osteoporosis is generally poor. Within six to 12 months of starting therapy, 20-30% of patients receiving daily, or weekly treatments may decide to stop receiving them. It is reported that patients at risk for hospitalization and osteoporotic fractures have low adherence. The majority of individuals who stop their medication tend to do so due to negative side effects. Another frequently mentioned justification for stopping therapy is fear of side effects or other health hazards. Some factors associated with adherence to medications among patients include fractures, fewer non-osteoporosis drugs, and co-morbidities, early menopause, willingness to take medications, knowledge of osteoporosis status based on a diagnostic test, anti-inflammatory therapy, and corticosteroid therapy while factors contributing to non-adherence include uncertainty about the results of the BMD test, adverse reactions, and pain [25]. Reginster narrated that it has been observed that adherence rates to oral bisphosphonate therapy decrease significantly during the first year of treatment and continue to decline thereafter. An increased risk of fracture along with slower or gradual changes in BMD is associated with a lack of medication adherence [26]. Our study's results align with existing literature, which reports low compliance rates in the majority of studies. Only three of the studies included reported optimal adherence rates. However, our review has also revealed conflicting results, as two of the included studies showed higher rates of fractures among the adherent population.

Varenna and Sinigaglia described that evidence suggests that pharmacological non-adherence affects more people than those with certain diseases or medications. Increased fracture risk, greater resource utilization, and hospitalization have all been linked to poor adherence. Treatment non-adherence for osteoporosis has uncertain origins. Older age, comorbidity, prior fractures, BMD evaluation, number of prescriptions, and institutionalization are some characteristics predictive of non-adherence, however, they account for a lesser percentage of the variability in adherence. Patients may stop taking their prescriptions for a variety of reasons, such as annoyance or difficulty with the dose, excessive cost, adverse effects, and a lack of understanding of the therapeutic advantages [27]. Likewise, Siris et al. described that fragility fracture rates are increasing in reality due to low rates of adherence and persistence to osteoporosis treatments. This highlights the significance of good treatment adherence and perseverance with osteoporosis medications in order to obtain a broader therapeutic benefit and therefore lessen the financial and social burden that osteoporosis and related fractures inflict on individuals and healthcare systems [28].

Similarly, Caro et al. reported that an almost 16% decrease in fracture rates was observed in complied patients. After adjusting for additional patient factors that independently predict the fracture rate, this association remained apparent within subgroups. Hence, the authors recommended that increasing compliance in real-world settings could dramatically reduce the incidence of fractures caused by osteoporosis [29]. Another study by Huybrechts, Ishak, and Caro revealed that at complete follow-up, 75% of study participants had a medication possession ratio lower than 80%. After accounting for other recognized risk variables, low compliance was linked to a 17% increase in the fracture rate. Additionally, low compliance was linked to a 37% increase in the likelihood of hospitalization for any reason, as well as higher average monthly costs for all medical services included (P <0.0001). When considering the gradients of compliance, similar relationships were observed [30]. Results of a comparative study showed that compliant women (medication possession ratio > 80%) had a 21% reduced total fracture risk than non-compliant women, Additionally, in women taking bisphosphonates, the probability of fracturing a bone began decreasing only at medication possession ratio levels of about 50%, and it continued to do so as compliance increased up to 90-100% [31]. Our results also show a similar trend with respect to fracture rates, the rates of fractures were decreased with adherent osteoporotic therapy except for two studies that showed increased fracture rates among the adherent study participants and two studies included also showed very slight changes in results between both groups of the adherent and non-adherent population.

Poor adherence to anti-osteoporosis medication is linked to decreased efficacy of these treatments, which can result in fractures, higher medical costs, and mortality. Additionally, a previous study demonstrated that better osteoporosis drug compliance can lower osteoporosis-related medical expenses by reducing fractures [32]. Similarly, Bonafede et al. reported that when compared to individuals with poor adherence, people with higher osteoporosis drug adherence showed a 28-32% lower risk of fracture [33]. Results of a meta-analysis revealed that with non-compliance, the absolute frequency of fracture ranged from 6% to 38%, and with non-persistence (104-159 weeks), it ranged from 5% to 19%. Non-compliance raises the risk of fracture by around 30% and non-persistence increases the risk by 30% to 40%. Compared to ideal adherence, poor medication compliance is linked to a considerably higher risk of fracture. Therefore, increasing compliance with medications in osteoporosis patients may result in a higher decrease in fractures [34]. Results of another meta-analysis concluded that for postmenopausal women receiving bisphosphonate treatment for osteoporosis, persistence, and compliance are poor. A higher likelihood of fracture, which is smaller for non-vertebral fractures than for clinical vertebral fractures, is the clinical repercussion of this inadequate compliance [35].

Results of an observational analysis depicted that optimal adherence levels were observed in nearly more than half of the patients, More than one-third of the patient population had low adherence to osteoporosis treatments. Patients with low and moderate adherence were 33% and 19% more likely, respectively, to experience subsequent fractures compared to those with high adherence, even after adjusting for demographic and clinical factors. When low-adherence patients were compared to high-adherence patients, the risks of non-vertebral and clinical vertebral fractures were increased by 32% and 34% respectively. Women who had low and moderate adherence to osteoporosis treatments had a higher risk of suffering a future fracture when compared to those with high adherence [12]. Despite the fact that osteoporosis medicine considerably lowers the risk of fractures, medication adherence is low among patients. Drug expenses are higher for medication adherents than for non-adherents, but adherence can reduce disease-related medical costs related to fractures, such as inpatient care and surgery [18]. Lower fracture rates among high adherent participants versus low or moderate adherent individuals were also observed in our study results.

Findings of a case-control study demonstrated that patients with >180 days of therapy had a significantly decreased fracture risk than those with or=30 days of therapy. Additionally, risk was decreased for individuals with a medication possession ratio of >90% compared to 30%. As compliance increased, the probability of fracture reduced (p< 0.05). A lower risk of fracture is linked to higher compliance among women starting medication therapy for osteoporosis [36]. Emkey and Ettinger stated that in accordance with the evidence, individuals with osteoporosis who take their medications consistently over a lengthy period of time have a significantly decreased risk of fracture. Better BMD and enhanced suppression of bone turnover indicators are also associated with therapy adherence. Despite being the strongest antiresorptive medicines that are currently licensed, bisphosphonates have unique dosage difficulties that can have a negative influence on long-term persistence, and adherence to weekly regimens is still not at its best [37]. Three studies included in our review discussed changes in terms of the effect of adherence on BMD with one reporting minor changes and the other stating significant changes among the adherent group.

Liu et al. described adherence to osteoporosis treatment considerably reduces the risk of fractures as demonstrated by several clinical However, in real-world settings, treatment adherence is frequently suboptimal, which can diminish the effectiveness of the intervention [38]. The results of our study emphasize the significance of medication adherence for decreasing the fracture risk and effectively treating osteoporosis. However, the included studies showed variation in fracture rates, with two studies reporting opposing findings. Moreover, only three studies described the effect of medication adherence on BMD. Therefore, further clinical research, including meta-analyses and randomized clinical trials, is needed to confirm and generalize the impact of medication adherence on fracture rates and BMD in individuals with osteoporosis. The limited literature on this topic further underscores the necessity for such research.

The present systematic review has some limitations. Firstly, the studies included in this review were diverse in terms of study design, patient population, and outcome measures, which may limit the ability to draw firm conclusions. Secondly, some studies reported on fracture risk while others reported on BMD, which could have introduced heterogeneity in the findings. Thirdly, not all studies included in the review may have controlled for all potential confounding factors, such as age, sex, comorbidities, and lifestyle factors, which could have affected the validity of the results. Overall, it is important to acknowledge these limitations when interpreting the findings of this systematic review.

## Conclusions

Diverse rates of fractures were observed with decreased rates of fracture reported in the adherent population however two of the included studies showed contradictory results. The findings of our study further highlight the importance of medication adherence among osteoporotic patients although the variation observed in our findings advocates the need for further research for the generalizability of results. Osteoporotic patients shall be counseled and educated by healthcare staff for effective adherence to pharmacological treatment to achieve optimal outcomes.
